# Pediatric Lisfranc Fracture-Dislocation: A Case Report

**DOI:** 10.7759/cureus.29525

**Published:** 2022-09-24

**Authors:** Moisés Ventura, Andreia Ferreira, Domingues Rodrigues, Raul Cerqueira, Mafalda Santos

**Affiliations:** 1 Ortopedia e Traumatologia, Centro Hospitalar Vila Nova de Gaia-Espinho, Vila Nova de Gaia, PRT; 2 Ortopedia e Traumatologia, Centro Hospitalar Vila Nova de Gaia/Espinho, Vila Nova de Gaia, PRT

**Keywords:** trauma, foot, pediatric, fracture-dislocation, lisfranc injury

## Abstract

Lisfranc injury is extremely rare in the pediatric population and little evidence exists to guide the treatment at this age.

We present a clinical case of a rare Lisfranc fracture-dislocation at pediatric age. An 11-year-old male was admitted to the emergency department, in October 2020, after a motorcycle incident. He was diagnosed with a Lisfranc fracture-dislocation of the right foot: Myerson type B2. Fourteen days after the injury, he underwent surgical treatment with open reduction and internal fixation with 3.5 mm solid fully threaded screws.

At 18 months postoperative, the patient was asymptomatic, didn’t present any limitations, presented an American Orthopedic Foot and Ankle Score (AOFAS) midfoot score of 93%, and excellent results of the 12-Item Short Form Survey (SF-12) - PCS-12 (Physical Score): 52.52277 and MCS-12 (Mental Score): 62.12820. The foot maintained a good configuration without significant malalignment, however, a screw breakage occurred before the implant removal, and a premature physeal arrest developed on the base of the first metatarsal bone.

Clinical and radiographic evaluation of Lisfranc injuries may be challenging in the pediatric population. Regarding the treatment, anatomical alignment is mandatory, and good or excellent outcomes have been achieved with anatomical reduction and internal fixation. We recommend early implant removal to avoid screw breakage and avoid the use of screws in the first metatarsal physis, due to the risk of premature physeal arrest.

## Introduction

The term ‘Lisfranc injury’ refers to an injury in which one or more of the metatarsals are displaced from the tarsus. This term was attributed in honor of a French surgeon of the Napoleonic era who first described this injury in 1815, describing also an amputation at that level [1]. 

The use of the term 'Lisfranc injury' is very broad and may be associated with a variety of presentations. These lesions range from purely ligamentous injuries to fractures with no ligamentous disruption and even a combination of the two [1-3].

They are infrequent injuries, corresponding to approximately 0.2% of all fractures. According to the literature, they are also frequently misdiagnosed, in which 20% of cases are not diagnosed or have a late diagnosis [1,2]. 

The mechanisms of injury, that are commonly described, are very similar in both Pediatric and adult reports of Lisfranc injuries. Mostly they occur from a direct plantar force (e.g. crush injury) or an indirect rotational force with the foot in plantar flexion (twisting injury) [1-4].

In children, Lisfranc injuries are extremely rare and little evidence exists to guide the diagnosis and management in the pediatric population. A reduced number of case series exist in the pediatric orthopedic literature [2,4-12], the largest article includes only 56 patients [2]. These injuries in the pediatric population are not only rare but also present similarities to adult Lisfranc injuries, they are frequently misdiagnosed or overlooked. In addition, it is important to treat them with an anatomic reduction to prevent complications [2]. When left untreated, they may predispose to midfoot degenerative arthritis, disability, and chronic pain [7,8]. 

We present a clinical case about a rare Lisfranc fracture-dislocation in pediatric age, treated with open reduction and internal fixation (ORIF).

## Case presentation

An 11-year-old male was admitted to our emergency department, in October 2020, after a motorcycle incident (direct trauma of the foot on the road after a fall). He mentioned pain in the right foot and inability to bear weight. On physical examination, he presented marked swelling throughout his midfoot and pain on palpation over all tarsal-metatarsal joints. There were no signs of plantar ecchymosis, compartment syndrome, or neurovascular injury.

**Figure 1:**
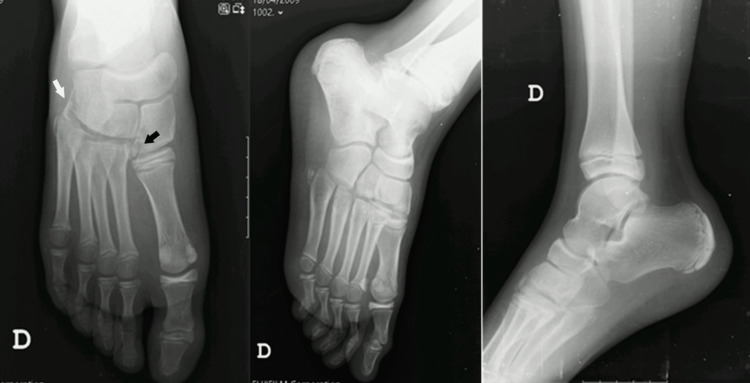
Initial X-rays of the right foot and ankle. Black arrow represents the fracture of the lateral aspect of the medial cuneiform (“fleck sign”); White arrow represents the fracture of the cuboid and subluxation of the M2-M5 tarsal-metatarsal joints.

X-rays of the right foot and ankle were requested showing a fracture of the lateral aspect of the medial cuneiform (“fleck sign”), fracture of the cuboid, apparent subluxation of the M2-M5 tarsal-metatarsal joints with the widening of the spaces between the first and second metatarsals (Figure 1).

**Figure 2:**
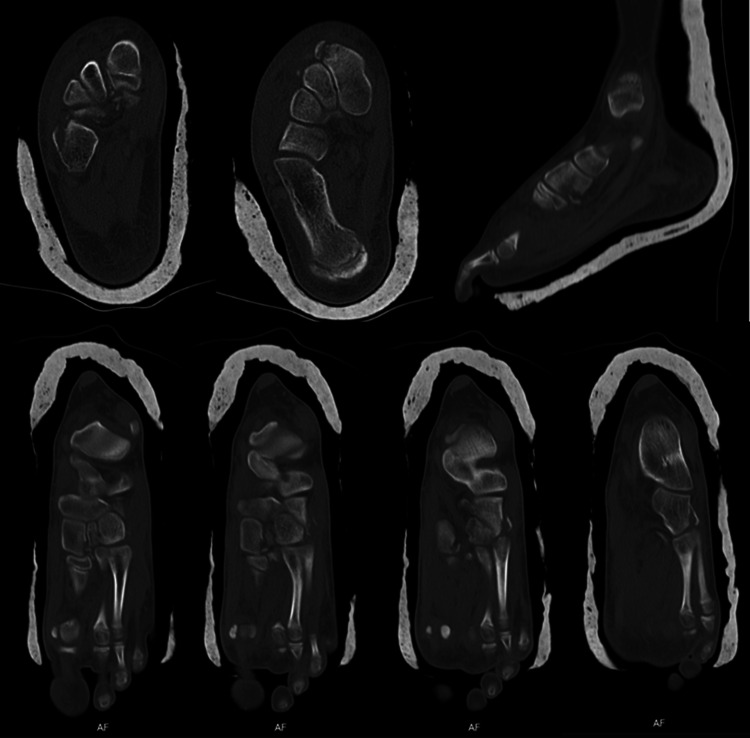
Computerized Tomography scan images.

A computerized tomography scan was requested to confirm the diagnosis and to plan the management of the injury. It revealed: “a comminuted fracture of the medial and distal face of the medial cuneiform, probably associated with rupture of the Lisfranc ligament with bone avulsion. There is also a fracture of the lateral cuneiform and cuboid, the latter with extensive periosteal detachment. There is also a register of an organizing hematoma deep in the extensor digitorum and hallux tendons.” (Figure 2).

Based on the clinical and radiographical findings he was diagnosed with a Lisfranc fracture-dislocation of the right foot - Myerson type B2.

**Figure 3:**
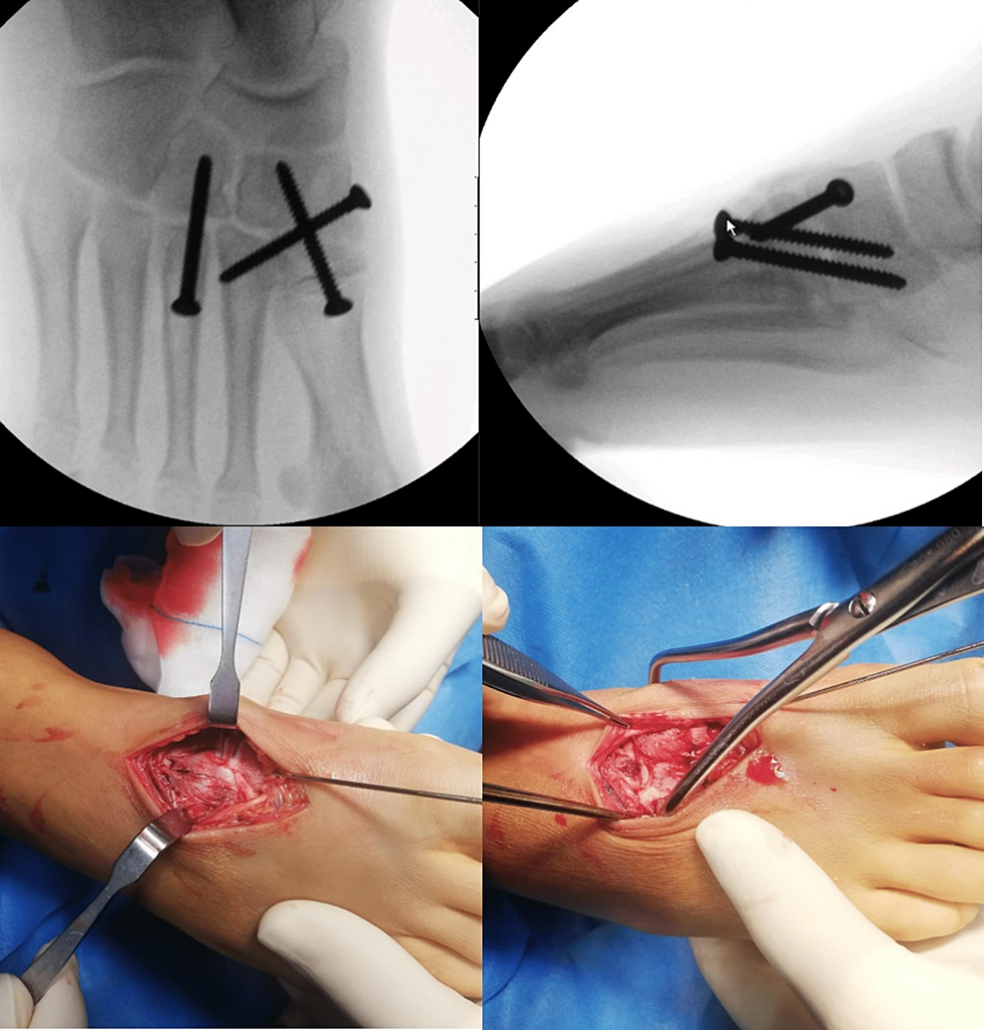
Intra-operative images.

Due to the swelling and the poor skin conditions, he was immobilized with a below-knee back slab, however, the definitive treatment was postponed, and a close follow-up was maintained until skin conditions were obtained. 14 days later, ORIF was done with 3.5 mm solid fully threaded screws - via a dorsal double parallel approach (longitudinal incision in the first inter-metatarsal space with another incision between the third and fourth metatarsal) (Figure 3).

Starting from medial to lateral, we reduced the first cuneiform-metatarsal joint and provisionally stabilized it with a K-wire, and later the definitive fixation was with a non-cannulated screw. The next step was to reduce the space between the first and second metatarsals. To this end, it was applied a reduction clamp between the medial area of the first cuneiform and the lateral area of the second metatarsal. The fixation was obtained using a non-cannulated screw from the first cuneiform to the base of the second metatarsal. Through the second incision (between the third and fourth metatarsal) the third tarsal-metatarsal joint was evaluated, reduced, and fixated with a 3.5 mm non-cannulated screw. After these maneuvers, we found that the joint between the cuboid and the fourth and fifth metatarsals, was reduced and stable not requiring further stabilization.

**Figure 4:**
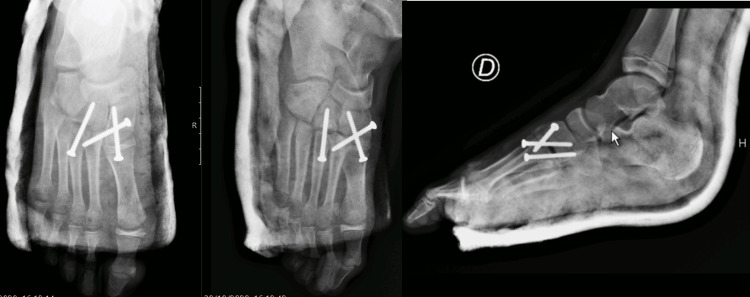
Postoperative X-rays.

He was immobilized with a below-knee back slab, the leg was kept elevated, and maintained close observations during the hospital stay. He was discharged home the following day without bearing weight (Figure 4).

Periodic clinical evaluations and X-rays were performed after two weeks, six weeks, and four months. He maintained immobilization for six weeks; after that period, progressive weight bearing was allowed and physiotherapy was started. No sports activities were allowed for four months.

**Figure 5:**
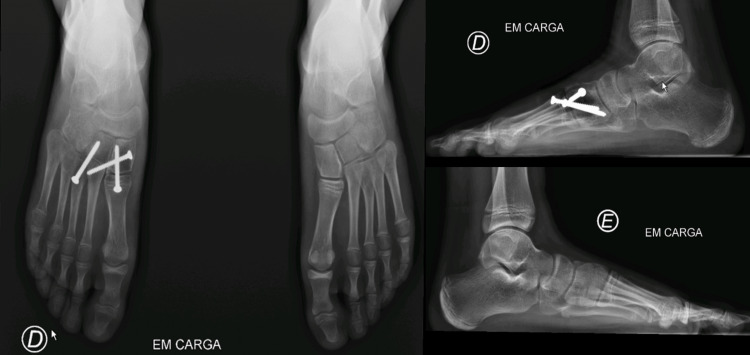
Weight-bearing bilateral X-rays: 4 months postoperative.

After four months, the patient was asymptomatic, with a normal range of motion, no limitations on daily activities, and radiographic signs of a union. So, removal of the osteosynthesis material was proposed at this stage (Figure 5).

The implant removal took place nine months post-operative, however, in the meanwhile, there was a screw breakage, which made it impossible for the total removal of the screw between the first cuneiform-metatarsal joint with retention of part of the screw in the medial cuneiform.

In the last observation, at 18 months post-operative, the patient was asymptomatic and did not present any limitations. Presented an AOFAS Midfoot score of 93% (excellent clinical result) and also excellent results on 12-Item Short Form Survey (SF-12) - PCS-12 (Physical Score): 52.52277 and MCS-12 (Mental Score): 62.12820 (Table 1).

**Table 1:** Functional outcome and quality of life score

		Before fracture	Follow-up 18months
AOFAS midfoot score		100%	93%
12-Item Short Form Survey (SF-12)	PCS-12 (Physical Score)	55.25834	52.52277
	MCS-12 (Mental Score)	60.69717	62.12829

**Figure 6:**
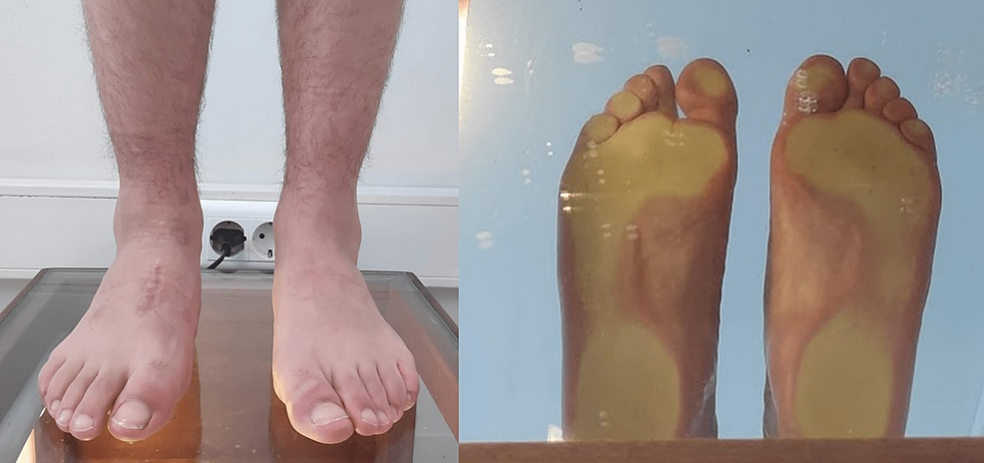
Foot morphology on the podoscope

The foot maintained a good configuration without significant malalignment, with a neutral Foot Posture Index-6 and a normal podoscopy (Figure 6). 

**Figure 7:**
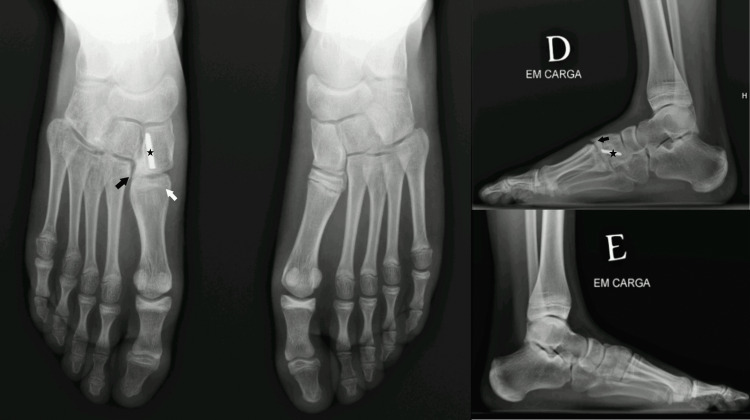
Weight-bearing bilateral X-rays: 18 months post-operative. The black arrow represents the spur on the medial cuneiform; the white arrow represents the premature physeal arrest on the base of the first metatarsal; Star represents the part of the screw-retained in the medial cuneiform.

Radiologically, a spur was present on the medial cuneiform, which may correspond to the calcification of the Lisfranc ligament. Additionally, although the good alignment was maintained, there was the development of a premature physeal arrest on the base of the first metatarsal with the shortening of this bone (Figure 7).

## Discussion

Lisfranc injuries are known to be difficult to diagnose [1,3,13]. Even though this point has not been directly referred to in the pediatric orthopedic literature, the lack of data on the matter may indicate that the entity is rare and/or underdiagnosed in this age group. The limited literature on pediatric Lisfranc injuries is mainly constituted by small case series. For that reason, it is challenging to counsel patients and parents on the management of these injuries [2].

The key to successful management is the determination of whether to use surgical stabilization [3]. No matter the severity of the lesion, the objective of treatment is a painless, plantigrade, and stable foot. The critical factor seems to be related to obtaining an anatomical alignment, which leads to a greater probability of a satisfactory result, but it does not guarantee it [3]. In addition, in the pediatric population, the management of these lesions may also be conditioned by skeletal maturity to some extent [2]. 

Analyzing the literature, in the largest case series described, Hill et al. assessed 56 children that were treated for Lisfranc injuries (bony and ligamentous) with ORIF or with conservative treatment. 77% of the patients with open physes underwent conservative treatment, while 67% of patients with closed physes were surgically treated. No statistical correlation was found between age and type of injury, but a large percentage of patients presented displaced injuries that required surgery (a total of 34% of the cohort), even if they had open physes [2].

In another study, Veijola et al. conducted a retrospective study that included six patients (aged between 13 and 16 years) surgically treated with ORIF. In all but one case, the anatomical reduction was achieved, but the majority of the patients (85,7%) claimed discomfort in the injured foot [5]. 

Kushare et al. evaluated 30 patients with a mean age of 13.6 years and a mean follow-up of 36 weeks. In their study, it was observed that 20% of the cases were missed on the initial presentation. Regarding the management, 19 cases were treated operatively while 11 were subjected to conservative treatment. Indications for surgery were > 2 mm displacement on weight-bearing radiographs and/or the presence of ‘fleck sign’ avulsion fracture on radiographs/advanced imaging, suggesting possible instability. The average Oxford Ankle Foot Questionnaire for Children (OxAFQ-C) and Visual Analogue Scale (VAS) pain scores obtained were 83% and 1.3 respectively, with a mean follow-up of 36 weeks. However, in terms of functional outcomes, no statistically significant differences were found between conservative and operative cases or between patients with isolated Lisfranc lesions and those with associated foot injuries [6]. 

This study emphasized the common misdiagnosis of Lisfranc lesions. For that reason, in suspected Lisfranc injuries that cannot be confirmed by plain radiographs, the use of weight-bearing views, computer tomography (CT), or magnetic resonance imaging (MRI) of the foot should be considered [1,3,13]. In our case, although the diagnosis could be established by the X-rays, the CT scan was important to recognize the displacement of the fracture, the presence of the ‘fleck sign’ avulsion fracture, and the subluxation of the lateral column with the cuboid’s fracture. This information was essential and provided the criteria required for surgical treatment with ORIF.

Regarding the type of fixation, recently, the suture button technique was developed as an alternative that provided the advantage of no need for a second surgery for hardware removal. Cardile et al. and Tzatzairis et al. reported cases of ligamentous injury treated with TightRopeTM in a 15-year-old male and an 11-year-old girl, respectively. The authors reported excellent results however the follow-up was very short. They concluded that the use of a dynamic fixation device for ligamentous Lisfranc in young patients can be a valid choice [9,10].

In our case, considering that there was a fracture-dislocation, we believed that ORIF with non-cannulated screws provided a better fixation with higher resistance to deformation. However, recent biomechanical studies have shown that fully threaded solid cortical and partially threaded cannulated screws provide equal amounts of fixation strength during partial weight bearing and similar resistance to deformation under bending loads. Partially threaded cannulated cancellous screws may simplify the operative procedure and minimize nonoptimal screw placement, so they can be used if the clinician so desires [14].

Another issue with using ORIF with screws passing the proximal physis of the first metatarsal is the physeal damage that may occur, compromising the correct growth of the bone. According to Scheuer and Black (2004), metatarsal fusion usually occurs between 14 and 16 years of age [15]. For that reason, the positioning of this screw may lead to premature physeal closure and concomitant shortening or deformity of the first metatarsal. In fact, this happened in our case, with the premature physeal arrest of the first metatarsal base presented at the 18th month of post-operative X-rays. Although the patient is asymptomatic, this deformity produces an Index Minus foot morphology, which is a known risk factor for Hallux Valgus and Metatarsalgia. A longer follow-up is needed to evaluate the evolution of the foot alignment and the clinical consequences of the first metatarsal shortening.

To avoid this complication, we believe that in skeletally immature patients (under 14 years old), the use of a Kirshner wire instead of a screw, is a valid alternative for the medial column fixation, if the required stabilization is obtained. This may cause a lesser damage area on the proximal physis and prevent the development of premature physeal arrest on the first metatarsal. In the alternative, dorsal plates with screws that do not cross the joint or the physes can also be used [1]. These plates provide stability without compromising the cartilage. In a biomechanical study in adults, comparing osteosynthesis with dorsal plates and transarticular screws, both methods had similar efficacy in reducing and resisting the displacement of the tarsal-metatarsal joint on weight-bearing [16].

Regardless of the type of fixation, the goal of the surgery is to obtain an anatomic alignment. Good or excellent results have been achieved in 50% to 95% of patients with anatomic alignment, compared with only 17% to 30% when the nonanatomic alignment was obtained [3]. In our case, the excellent outcome was due to the anatomical reduction of the fracture, which provided the correct alignment for the proper function of the foot. 

However, in some cases, even if the proper reduction is achieved, Lisfranc injuries may cause midfoot degenerative arthritis, chronic pain, and disability. Lesko et al. presented a case of a 10-year-old girl who experienced a Lisfranc fracture-dislocation in 2013. The injury was treated with ORIF and fixated with Kirshner wires, five years post-operatively she developed functional pain and radiographic signs of degenerative arthritis [8]. In our case, the patient was asymptomatic, with excellent functional outcomes and radiologically there were no evident signs of degenerative arthritis. Nevertheless, it was noted a spur in the medial cuneiform. This may correspond to calcification of the Lisfranc ligament, but we cannot exclude that it can be an early sign of a degenerative process. 

Regarding postoperative care, it seems to exist consensus about the need for protection of the reduction and the fixation for a minimum of 6 weeks [17]. In a systematic review, by Stavlas et al, screw problems were among the most common complications reported, making it necessary the removal of broken or problem-causing screws [6,17]. In fact, in our case, due to the prolonged time to remove the screws, one complication was the screw breakage, which made it impossible for the complete removal of the screw between the first cuneiform-metatarsal joint with the retention of part of the screw in the medial cuneiform. This might be another of the key points of our case, we recommend the screw removal around 12-16 weeks postoperative, to avoid screw breakage. This timing is presented, according to the literature, for an ideal ratio between bone healing and the potential for hardware breakage. The timing for hardware removal is very variable depending on the author and the hardware itself. For adults, it is established that K-wire fixation is usually removed at six weeks postoperatively, whereas screws are left in place for four months [3]. In the pediatric population, the timing for hardware removal is more variable [6,8,11]. In the study conducted by Kushare et al., the hardware (K-wires or screws) was removed after 28.5 days on average (range: 6-65), however, the authors did not present the specific timings for each type of hardware [6]. Concerning the complications associated with the retention of part of the screw, we should consider that any retained hardware constitutes a potential site for an implant-related infection, as the local immuno-incompetency creates an advantageous loci for infection [18]. Additionally, If part of the screw remains inside the joint (and not completely inside the bone), it may damage the cartilage and increases the risk for midfoot degenerative arthritis. By removing the hardware around 12-16 weeks postoperative, we follow the recommendations for adults, which provide reliability in the healing process and remains an early removal that prevents screw breakage and its complications [3]. 

## Conclusions

In the pediatric population, clinical and radiographic evaluation and management of Lisfranc injuries may be very challenging. Considering the high percentage of overlooked cases, a high index of suspicion is the key to diagnosing these injuries. Regarding the treatment, the goal is to obtain an anatomic alignment. Good or excellent results can be achieved with the anatomical reduction of the fracture and internal fixation.

Care should be taken using screws in the first metatarsal physis, this may cause a premature physeal arrest and an alteration of the foot morphology. We recommend screw removal once the fracture is consolidated to avoid screw breakage.
